# Sociodemographic differences in reasons for ENDS use among US youth within Wave 2 of the PATH study

**DOI:** 10.18332/tid/99879

**Published:** 2019-01-21

**Authors:** Connie Xiao, Kathryn Heley, Ryan David Kennedy, Lisa Lagasse, Meghan Bridgid Moran

**Affiliations:** 1 Johns Hopkins University, Baltimore, United States; 2 Department of Health Policy & Management, Johns Hopkins Bloomberg School of Public Health, Baltimore, United States; 3 Institute for Global Tobacco Control, Johns Hopkins Bloomberg School of Public Health, Baltimore, United States; 4 Department of Health, Behavior & Society, Johns Hopkins Bloomberg School of Public Health, Baltimore, United States

**Keywords:** ENDS, e-cigarettes, adolescents, motivations

## Abstract

**INTRODUCTION:**

Adolescents use electronic nicotine delivery systems (ENDS, or e-cigarettes) more than other tobacco products. Among adults, some data indicate that motivations for use vary by sociodemographic group. This study sought to examine how adolescents’ motivations for ENDS use vary by sociodemographic characteristics, including age, gender, race/ethnicity and household income.

**METHODS:**

The current study used data from Wave 2 of the Population Assessment of Tobacco and Health (PATH) study. Youth who used ENDS in the past 30 days were asked to report their motivations for product use. Rates of reporting each reason for use were compared across sociodemographic groups.

**RESULTS:**

Appealing flavors was the most commonly reported motivation for using ENDS, and was mentioned more often among females (89.23%) than males (74.00%). Females were also more likely than males to report using ENDS because the product feels like smoking cigarettes (AOR=1.761) and people who are important to the participant smoke them (AOR=1.895). Older teens were more likely to report using ENDS because the product does not smell bad (56.45%, 15–17 years old; 42.83%, 12–14 years old).

**CONCLUSIONS:**

Motivations for ENDS use vary by sociodemographic group. Understanding the motivations for use among sociodemographic subgroups is an initial step towards informing the development of policies and interventions with equally distributed benefits.

## INTRODUCTION

Recent data indicate that American youths’ use of electronic nicotine delivery systems (ENDS) exceeds that of other tobacco products^1^. As of 2017, 11.7% of high schoolers and 3.3% of middle schoolers used ENDS in the past 30 days^1^. Research examining sociodemographic differences in ENDS use indicates that males^2^, adolescents of higher socioeconomic status^3^, and non-Hispanic White youth^4^ may be more likely to use ENDS. Although ENDS have been positioned as potential harm reduction tools for combustible cigarette smokers^5^, their use among adolescents remains problematic as ENDS solutions and aerosols contain numerous toxicants and carcinogens^6^, in addition to nicotine, an addictive substance with neurotoxic effects on the developing brain^7^.

Studies have identified several reasons for ENDS’ increasing popularity among adolescents, including greater visibility in the media^8^, combustible cigarette smoking cessation^9^, interest in flavors^10^, and peer influence^11^. Like other tobacco products^12^, motivations for ENDS use may vary with sociodemographic characteristics. Studies of US adults demonstrate that appealing flavors are more likely to be a reason for use among young adults^13^, and adult women are more likely to report using e-cigarettes because of their use by friends or family members^14^.

Limited research has examined differences in motivations for ENDS use among sociodemographic subgroups of adolescents. A clear understanding of these variations is valuable to help ensure that messages, interventions and policies are relevant to populations most vulnerable to ENDS use and are culturally tailored as needed. This study examines how adolescents’ motivations for ENDS use vary by sociodemographic characteristics, including age, gender, race/ethnicity and household income.

## METHODS

### Data

We analyzed data from the Wave 2 youth sample of the Population Assessment of Tobacco and Health (PATH) study (N=12172)^15^. PATH was a longitudinal cohort study of US youth and adults and its Wave 2 data collection occurred between 2014–2016. Further study design and sample details are described in Hyland et al.^15^ and the study questionnaire can be found at https://www.icpsr.umich.edu/icpsrweb/NAHDAP/studies/36231. Our sample for analysis consisted of 415 youth participants who were asked questions about their motivations for ENDS use. These questions were only asked to participants who indicated they had used ENDS in the past 30 days. PATH study procedures were approved by the Westat Institutional Review Board^15^.

### Measures

The primary measures of interest were questions asking youth to identify, from a list of 13 possibilities, the reasons they use ENDS (response options of yes/ no). Table 1 presents the full wording for each item. We examined how motivations varied across several sociodemographic characteristics available in the PATH Public Use File: age (12–14, 15–17 years), gender (female, male), race/ethnicity (Hispanic, non-Hispanic Black, non-Hispanic White, and other non-Hispanic ethnicity), and household income (obtained from the youth’s parent: <$10000, $10000–$24999, $25000–$49999, $50000–$99999, ≥$100000).

**Table 1:** Sample characteristics, United States, 2014–2016 (N=415 )

	*Per cent*	*95% CI*	*N (unweighted)*
**Age** (years)			
12–14	19.59	(15.65–24.24)	88
15–17	80.41	(75.76–84.35)	327
**Gender**			
Male	56.96	(51.53–62.24)	237
Female	43.04	(37.76–48.47)	177
**Race**			
Non-Hispanic White	69.34	(64.24–74.01)	264
Non-Hispanic Black	5.55	(3.50–8.68)	19
Non-Hispanic Other	8.28	(5.84–11.61)	42
Hispanic	16.83	(13.67–20.56)	90
**Household income**			
Less than $10000	8.11	(5.57–11.65)	31
$10000–$24999	16.82	(13.18–21.22)	75
$25000–$49999	20.35	(16.17–25.29)	76
$50000–$99999	29.96	(25.32–35.05)	110
$100000 or more	24.77	(19.56–30.83)	84
**Used flavored e-cigarette in past 30 days**			
Yes	79.36	(74.65–83.39)	329
No	10.92	(8.2–14.39)	42
Don't know	9.72	(7.18–13.03)	43
**Current combustible tobacco user**			
Non-combustible at W1 and W2	46.12	(40.95–51.38)	181
Combustible at W1 only	5.5	(3.63–8.27)	22
New W2 combustible (combustible at W2 only)	23.83	(18.95–29.52)	95
Current combustible use at W1 and W2	24.54	(20.11–29.58)	103
**Days used e-cigarette in past 30 days**^a^	7.751	(6.831–8.672)	
**Reasons for e-cigarette use**			
1. It comes in flavors I like	77.90	(73.07–82.07)	327
2. They might be less harmful to me than cigarettes	75.01	(70.09–79.37)	306
3. They might be less harmful to people around me	74.08	(68.61–78.90)	307
4. It helps people to quit smoking cigarettes^b^	63.48	(57.22–69.32)	162
5. They can be used in places where smoking can’t	58.68	(53.24–63.92)	242
6. They don't bother non-tobacco users	56.40	(50.74–61.91)	229
7. They don't smell	53.80	(48.58–58.93)	229
8. They are affordable	48.18	(43.91–52.48)	208
9. I like socializing while using them	45.80	(41.64–50.01)	193
10. People in the media or other public figures use them	36.11	(30.91–41.65)	150
11. People who are important to me use them	34.88	(29.69–40.46)	143
12. It feels like smoking a regular cigarette	25.09	(20.31–30.57)	107
13. The advertising appeals to me	14.18	(10.63–18.66)	60

a Sample restricted to youth who are past 30-day e-cigarette users.

b Asked to youth who last smoked a cigarette within the past year.

### Data analysis

We used the weighting and variance estimation procedures provided in the PATH user guide. We calculated descriptive statistics to characterize our sample, then computed crosstabs, with chi-squared tests of significance, to examine bivariate differences in the prevalence of reported motivations for use across identified sociodemographic characteristics. We conducted logistic regression analyses to examine unique associations between sociodemographics and motivations for ENDS use, entering all sociodemographic variables together in models for each motivation, controlling for current combustible tobacco use, intensity of ENDS use (number of days used in past 30 days) and whether the participant uses a flavored ENDS. Analyses were conducted using Stata 14.

## RESULTS

Table 1 presents full descriptive statistics for the analytic sample. The most commonly selected reasons for ENDS use were: appealing flavors (77.9%), ‘they might be less harmful to me than cigarettes’ (75.0%) and ‘they might be less harmful to people around me’ (74.1%). Bivariate analyses revealed several differences across sociodemographic characteristics in motivations for ENDS use (Table 2). Among females, 82.9% reported using ENDS because the product comes in appealing flavors (compared to 74.0% of males), 31.3% reported using ENDS because the product feels like smoking cigarettes (compared to 20.5% of males), and 43.3% reported using ENDS because people who are important to them use them (compared to 28.2% of males). Just over half (53.6%) of non-Hispanic Black youth reported use due to appealing flavors, compared to non-Hispanic Whites (78.8%), Hispanics (76.6%), and those of other non-Hispanic ethnicities (89.3%). Among older adolescents (15–17 years old), 56.5% reported using ENDS because the products do not smell bad, compared to 42.8% of younger adolescents (12–14 years old). Among those with household incomes of less than $10000 a year, 16.3% reported using ENDS because the advertising appealed to them, compared to 26.4% of those with household incomes of $10000– $24999, 15.0% of those with household incomes of $25000–$49999, 11.5% of those with household incomes of $50000–$99999 and 4.34% of those with household incomes of $100000 or more.

**Table 2:** Bivariate differences in e-cigarette reasons for use by sociodemographic factors, United States, 2014– 2016 (N=415 )

	*It comes in flavors I like*			*They might be less harmful to me than cigarettes*			*They might be less harmful to people around me*		
	*N*	*%*	*95% CI*	*N*	*%*	*95% CI*	*N*	*%*	*95% CI*
**Overall**	327	77.85	(73.01–82.03)	306	74.96	(70.02–79.33)	307	748.00	(68.61–78.90)
**Age** (years)									
12–14	71	79.99	(70.71–86.88)	69	80.02	(69.78–87.42)	68	79.32	(68.17–87.29)
15–17	256	77.38	(71.70–82.21)	237	73.81	(68.04–78.86)	239	72.83	(67.21–77.80)
**Gender**									
Male	**179**	**74.00**	(67.79–79.38)*	175	75.54	(68.57–81.39)	174	72.51	(64.65–79.18)
Female	**147**	**82.93**	(76.02–88.15)*	130	74.20	(67.71–79.78)	132	76.04	(68.97–81.93)
**Race/Ethnicity**									
Non-Hispanic White	**210**	**78.79**	(72.88–83.70)*	196	74.52	(68.31–79.87)	193	72.02	(65.40–77.81)
Non-Hispanic Black	**11**	**53.62**	(27.28–78.09)*	16	89.57	(67.44–97.27)	14	81.03	(59.06–92.67)
Other race	**37**	**89.30**	(75.69–95.72)*	32	78.90	(62.72–89.27)	33	82.36	(65.44–92.01)
Hispanic	**69**	**76.59**	(65.42–84.98)*	62	70.20	(58.85–79.52)	67	76.32	(65.61–84.49)
**Household Income**									
<$10000	22	71.82	(53.87–84.77)	22	68.38	(49.20–82.85)	22	71.35	(49.16–86.51)
$10000–$24999	59	79.10	(68.54–86.79)	55	79.79	(71.14–86.35)	54	71.80	(55.73–83.73)
$25000–$49999	62	79.42	(67.52–87.75)	55	73.00	(61.16–82.27)	60	78.16	(67.16–86.23)
$50000–$99999	86	77.94	(68.67–85.06)	76	71.06	(61.46–79.08)	79	71.29	(61.95–79.10)
$100000 or more	68	77.26	(61.90–87.67)	66	76.47	(63.46–85.88)	61	72.53	(59.63–82.51)

**Table:** 

	*It helps people to quit smoking cigarettes*			*They can be used in places where smoking can’t*			*They don’t bother non-tobacco users*		
	*N*	*%*	*95% CI*	*N*	*%*	*95% CI*	*N*	*%*	*95% CI*
**Overall**	162	63.48	(57.22–69.32)	242	58.81	(53.36–64.05)	229	56.31	(50.65–61.81)
**Age** (years)									
12–14	31	64.75	(50.33–76.91)	51	60.11	(48.71–70.51)	45	52.41	(41.32–63.26)
15–17	131	63.20	(56.76–69.21)	191	58.33	(52.33–64.10)	184	57.39	(51.06–63.49)
**Gender**									
Male	96	68.70	(59.54–76.61)	145	60.81	(52.65–68.41)	133	57.54	(50.55–64.24)
Female	66	57.58	(48.27–66.39)	97	56.16	(49.32–62.77)	95	54.68	(45.90–63.18)
**Race/Ethnicity**									
Non-Hispanic White	100	60.51	(52.82–67.72)	155	60.00	(53.15–66.49)	146	56.09	(48.29–63.60)
Non-Hispanic Black	7	83.42	(48.10–96.47)	10	48.39	(22.39–75.28)	11	65.32	(40.20–84.07)
Other race	17	67.83	(42.71–85.64)	25	59.39	(42.56–74.27)	21	54.25	(36.62–70.88)
Hispanic	38	69.52	(55.02–80.96)	52	56.36	(45.67–66.49)	51	55.90	(45.63–65.69)
**Household Income**									
<$10000	13	77.36	(47.60–92.78)	14	44.26	(27.61–62.30)	15	45.71	(27.19–65.51)
$10000–$24999	39	68.42	(51.14–81.77)	49	68.39	(54.87–79.37)	44	59.42	(45.45–72.01)
$25000–$49999	35	63.61	(49.71–75.55)	43	55.76	(43.25–67.58)	43	56.12	(42.81–68.60)
$50000–$99999	37	60.43	(47.10–72.37)	62	57.04	(46.70–66.80)	61	54.85	(44.87–64.46)
$100000 or more	28	60.87	(44.98–74.75)	48	57.24	(43.17–70.23)	42	54.57	(40.90–67.59)

**Table:** 

	*They don’t smell*			*They are affordable*			*I like socializing while using them*		
	*N*	*%*	*95% CI*	*N*	*%*	*95% CI*	*N*	*%*	*95% CI*
**Overall**	229	53.70	(48.48–58.83)	208	48.07	(43.79–52.37)	193	45.68	(41.50–49.92)
**Age** (years)									
12–14	40	**42.83**	(32.72–53.58)*	43	46.30	(35.65–57.30)	34	37.20	(28.14–47.26)
15–17	189	**56.46**	(50.47–62.26)*	165	48.64	(43.28–54.02)	159	47.86	(42.56–53.20)
**Gender**									
Male	125	51.38	(44.84–57.88)	132	53.37	(46.46–60.17)	112	44.44	(38.31–50.73)
Female	103	56.75	(49.42–63.79)	75	40.96	(32.81–49.64)	80	47.33	(40.29–54.48)
**Race/Ethnicity**									
Non-Hispanic White	142	53.02	(45.85–60.06)	119	45.02	(39.89–50.26)	122	45.21	(40.31–50.21)
Non-Hispanic Black	9	41.57	(21.55–64.82)	8	36.85	(16.20–63.77)	6	30.63	(11.72–59.48)
Other race	28	66.84	(49.25–80.71)	28	63.18	(46.34–77.32)	17	44.34	(26.94–63.25)
Hispanic	50	54.48	(42.12–66.31)	53	57.53	(45.70–68.56)	48	53.96	(42.93–64.63)
**Household Income**									
<$10000	14	44.27	(25.15–65.25)	14	38.44	(22.45–57.40)	13	38.60	(22.29–57.96)
$10000–$24999	42	54.14	(41.75–66.03)	36	45.81	(33.11–59.08)	35	47.68	(35.85–59.77)
$25000–$49999	42	53.85	(41.28–65.94)	36	44.40	(32.73–56.73)	31	40.88	(29.70–53.08)
$50000–$99999	60	52.70	(44.02–61.22)	57	48.78	(39.51–58.14)	59	51.26	(40.70–61.71)
$100000 or more	51	58.30	(45.57–70.01)	43	50.76	(38.02–63.39)	39	43.73	(31.83–56.40)

**Table:** 

	*People in the media or other public figures use them*			*People who are important to me use them*			*It feels like smoking a regular cigarette*		
	*N*	*%*	*95% CI*	*N*	*%*	*95% CI*	*N*	*%*	*95% CI*
**Overall**	150	36.19	(30.98–41.74)	143	34.74	(29.56–40.31)	107	25.15	(20.35–30.64)
**Age (years)**									
12–14	39	45.10	(34.61–56.04)	31	34.86	(24.60–46.75)	23	25.68	(16.63–37.44)
15–17	111	33.93	(28.33–40.02)	112	34.89	(29.05–41.22)	84	24.94	(20.01–30.63)
**Gender**									
Male	86	34.78	(28.35–41.83)	67	**28.24**	(22.63–34.63)**	52	**20.49**	(15.68–26.32)*
Female	64	38.06	(29.98–46.86)	75	**43.28**	(35.62–51.27)**	55	**31.30**	(23.46–40.36)*
**Race/Ethnicity**									
Non-Hispanic White	88	33.91	(28.01–40.36)	98	36.12	(30.25–42.44)	67	24.70	(18.71–31.86)
Non-Hispanic Black	7	26.77	(9.28–56.62)	6	29.58	(13.18–53.75)	8	37.13	(18.64–60.35)
Other race	18	47.77	(30.13–65.99)	17	44.34	(28.13–61.85)	13	30.46	(17.98–46.68)
Hispanic	37	42.63	(32.96–52.91)	22	26.62	(18.62–36.51)	19	20.07	(13.59–28.61)
**Household Income**									
<$10000	13	39.31	(21.41–60.63)	8	26.72	(12.88–47.36)	9	**27.16**	(12.64–49.01)*
$10000–$24999	34	47.66	(35.99–59.59)	22	26.33	(17.31–37.91)	24	**32.86**	(22.84–44.73)*
$25000–$49999	28	38.73	(27.35–51.48)	25	34.49	(23.67–47.20)	22	**27.50**	(18.70–38.48)*
$50000–$99999	31	27.92	(19.44–38.34)	40	37.60	(29.24–46.77)	28	**28.15**	(19.64–38.57)*
$100000 or more	29	32.44	(22.35–44.49)	33	37.38	(26.71–49.45)	13	**13.80**	(8.58–21.46)*

**Table:** 

	*Advertising appeals to me*		
	*N*	*%*	*95% CI*
**Overall**	60	14.18	(10.63–18.66)
**Age (years)**			
12–14	20	21.50	(13.13–33.16)
15–17	40	12.37	(8.83–17.06)
**Gender**			
Male	38	14.45	(10.01–20.40)
Female	22	13.90	(9.02–20.80)
**Race/Ethnicity**			
Non-Hispanic White	35	13.02	(8.91–18.64)
Non-Hispanic Black	4	17.05	(5.21–43.48)
Other race	5	14.35	(5.44–32.76)
Hispanic	16	17.87	(10.69–28.35)
**Household Income**			
<$10000	**6**	**16.27**	(7.17–32.85)**
$10000–$24999	**18**	**26.42**	(16.65–39.22)**
$25000–$49999	**12**	**15.03**	(7.80–27.01)**
$50000–$99999	**13**	**11.45**	(6.65–19.03)**
$100000 or more	**4**	**4.34**	(1.56–11.51)**

* p<0.05

** p<0.01

*** p<0.001

N is unweighted, percentages are weighted. Cells may not sum to overall totals due to missing data.

Logistic regression analyses (Table 3) indicated that variations in use behavior were associated with several motivations for ENDS use. These analyses indicate that compared to non-Hispanic Whites, those of non-Hispanic ethnicities other than non-Hispanic White or Black had a higher likelihood of reporting using ENDS because they are affordable (AOR=2.684, 95% CI: 1.044–6.899), and females had a higher likelihood of reporting using ENDS because people important to them use ENDS (AOR=1.895, 95% CI: 1.128–3.184) and a lower likelihood of reporting using ENDS because they are affordable (AOR=0.538, 95% CI: 0.296–0.976). Combustible tobacco users had a higher likelihood of reporting using ENDS, because they compared favorably to cigarettes in that they do not smell, can be used in places cigarettes are not allowed, and feel like smoking a cigarette. Flavored ENDS users had a higher likelihood of reporting using ENDS due to the appealing flavors, reduced harm perceptions, and use in socialization or because people important to them use ENDS. Adolescents who use ENDS more intensely had a higher likelihood of reporting socialization, reduced harm to others, and not bothering other people as reasons for use.

**Table 3:** Adjusted odds ratios for association between sociodemographics and tobacco variables with motivations for e–cigarette use, United States, 2014–2016 (N=415 )

	*It comes in flavors I like*		*They might be less harmful to me than cigarettes*		*They might be less harmful to people around me*		*It helps people to quit smoking cigarettes*	
	*AOR*	*95% CI*	*AOR*	*95% CI*	*AOR*	*95% CI*	*AOR*	*95% CI*
**15–17 years old** (ref: 12–14 years old)	0.590	(0.291–1.198)	0.608	(0.295–1.25)	0.501	(0.244–1.030)	0.668	(0.308–1.449)
Female (ref: Male)	1.751	(0.928–3.305)	0.992	(0.577–1.707)	1.415	(0.781–2.564)	0.636	(0.337–1.202)
**Race/ethnicity** (ref: Non-Hispanic White)								
Non-Hispanic Black	0.512	(0.117–2.236)	4.818	(0.623–37.275)	2.594	(0.688–9.778)	4.221	(0.262–68.03)
Other race	1.875	(0.593–5.929)	1.309	(0.489–3.503)	2.093	(0.732–5.986)	1.702	(0.421–6.882)
Hispanic	1.000	(0.510–1.958)	0.648	(0.328–1.279)	1.287	(0.572–2.894)	1.174	(0.506–2.723)
**Household income** (ref: <$10000/year)								
$10000–$24999	1.385	(0.481–3.984)	1.757	(0.612–5.044)	0.838	(0.252–2.788)	0.625	(0.087–4.497)
$25000–$49999	1.516	(0.533–4.316)	1.116	(0.377–3.301)	1.188	(0.349–4.040)	0.435	(0.064–2.93)
$50000–$99999	1.222	(0.433–3.449)	1.025	(0.365–2.878)	0.773	(0.214–2.788)	0.402	(0.054–2.999)
$100000 or more	1.255	(0.388–4.061)	1.464	(0.441–4.857)	0.972	(0.255–3.704)	0.427	(0.056–3.277)
**Uses a flavored e-cigarette** (ref: does not use flavored e-cigarette)	**2.300**	(1.153–4.585)*	1.220	(0.642–2.316)	**1.976**	(1.082–3.605)*	1.978	(0.89–4.395)
**Combustible tobacco use during Wave 2** (ref: not during Wave 2)	1.416	(0.746–2.687)	1.525	(0.855–2.717)	1.337	(0.735–2.434)	1.275	(0.47–3.462)
**Days used e-cigarette in past 30 days**	1.035	(0.994–1.078)	1.025	(0.993–1.058)	**1.058**	(1.022–1.095)**	1.011	(0.976–1.047)

**Table:** 

	*They can be used in places where smoking can’t*		*They don’t bother non–tobacco users*		*They don’t smell*		*They are affordable*	
	*AOR*	*95% CI*	*AOR*	*95% CI*	*AOR*	*95% CI*	*AOR*	*95% CI*
**15–17 years old** (ref: 12–14 years old)	0.767	(0.398–1.478)	1.167	(0.649–2.098)	1.595	(0.829–3.069)	0.856	(0.463–1.584)
Female (ref: Male)	0.867	(0.524–1.435)	0.916	(0.548–1.563)	1.216	(0.752–1.965)	**0.538**	(0.296–0.976)*
**Race/ethnicity** (ref: Non-Hispanic White)								
Non-Hispanic Black	1.111	(0.271–4.550)	1.998	(0.610–6.552)	0.762	(0.221–2.630)	1.039	(0.234–4.610)
Other race	1.005	(0.386–2.619)	1.022	(0.440–2.374)	1.715	(0.699–4.208)	**2.684**	(1.044–6.899)*
Hispanic	0.835	(0.442–1.579)	0.913	(0.447–1.865)	1.632	(0.780–3.415)	1.934	(0.995–3.759)
**Household income** (ref: <$10000/year)								
$10000–$24999	2.221	(0.772–6.387)	1.837	(0.615–5.487)	1.457	(0.509–4.347)	1.119	(0.369–3.395)
$25000–$49999	1.192	(0.408–3.481)	1.420	(0.451–4.476)	1.635	(0.543–4.922)	1.186	(0.382–3.675)
$50000–$99999	1.345	(0.515–3.512)	1.280	(0.500–3.278)	1.361	(0.450–4.118)	1.412	(0.476–4.191)
$100000 or more	1.466	(0.512–4.198)	1.325	(0.522–3.364)	1.869	(0.572–6.110)	1.670	(0.527–5.299)
**Uses a flavored e-cigarette** (ref: does not use flavored e-cigarette)	1.663	(0.915–3.019)	1.252	(0.695–2.258)	1.586	(0.764–3.291)	1.827	(0.982–3.400)
**Combustible tobacco use during Wave 2** (ref: not during Wave 2)	**2.340**	(1.431–3.828)**	1.118	(0.714–1.751)	**1.740**	(1.117–2.711)*	1.483	(0.892–2.466)
**Days used e-cigarette in past 30 days**	**1.061**	(1.031–1.093)***	**1.037**	(1.004–1.070)*	1.012	(0.983–1.042)	1.023	(0.995–1.051)

**Table:** 

	*I like socializing while using them*		*People in the media or other public figures use them*		*People who are important to me use them*		*It feels like smoking a regular cigarette*	
	*AOR*	*95% CI*	*AOR*	*95% CI*	*AOR*	*95% CI*	*AOR*	*95% CI*
**15–17 years old** (ref: 12–14 years old)	1.247	(0.684–2.276)	0.741	(0.409–1.344)	0.922	(0.477–1.783)	0.802	(0.363–1.772)
Female (ref: Male)	1.212	(0.743–1.976)	1.193	(0.668–2.132)	**1.895**	(1.128–3.184)*	**1.761**	(1.060–2.927)*
**Race/ethnicity** (ref: Non-Hispanic White)								
Non-Hispanic Black	0.807	(0.188–3.464)	0.812	(0.212–3.101)	0.997	(0.307–3.236)	**2.740**	(1.000–7.513)*
Other race	0.967	(0.375–2.491)	1.966	(0.796–4.852)	1.494	(0.616–3.623)	0.801	(0.253–2.535)
Hispanic	1.581	(0.848–2.948)	1.197	(0.656–2.184)	0.657	(0.347–1.246)	0.674	(0.369–1.231)
**Household income** (ref: <$10000/year)								
$10000–$24999	1.438	(0.543–3.809)	1.118	(0.415–3.011)	1.011	(0.284–3.598)	1.556	(0.489–4.955)
$25000–$49999	1.042	(0.365–2.978)	0.904	(0.304–2.689)	1.513	(0.438–5.224)	1.142	(0.366–3.560)
$50000–$99999	1.426	(0.623–3.261)	0.521	(0.181–1.502)	1.444	(0.476–4.382)	1.310	(0.458–3.748)
$100000 or more	1.138	(0.391–3.312)	0.728	(0.244–2.166)	1.439	(0.463–4.476)	0.497	(0.168–1.467)
**Uses a flavored e-cigarette** (ref: does not use flavored e-cigarette)	**2.221**	(1.280–3.853)**	1.208	(0.64–2.281)	**3.005**	(1.532–5.892)**	0.947	(0.393–2.285)
**Combustible tobacco use during Wave 2** (ref: not during Wave 2)	1.157	(0.701–1.910)	1.177	(0.705–1.963)	0.874	(0.524–1.455)	**3.810**	(2.079–6.979)***
**Days used e-cigarette in past 30 days**	**1.033**	(1.003–1.064)*	1.006	(0.978–1.034)	1.012	(0.985–1.040)	1.008	(0.975–1.042)

**Table:** 

	*The advertising appeals to me*	
	*AOR*	*95% CI*
**15–17 years old** (ref: 12–14 years old)	0.488	(0.194–1.225)
Female (ref: Male)	0.807	(0.393–1.656)
**Race/ethnicity** (ref: Non-Hispanic White)		
Non-Hispanic Black	2.542	(0.523–12.348)
Other race	0.884	(0.154–5.091)
Hispanic	1.493	(0.662–3.368)
**Household income** (ref: <$10000/year)		
$10000–$24999	1.763	(0.380–8.168)
$25000–$49999	0.918	(0.210–4.006)
$50000–$99999	0.817	(0.223–3.000)
$100000 or more	0.289	(0.068–1.220)
**Uses a flavored e-cigarette** (ref: does not use flavored e-cigarette)	0.900	(0.423–1.913)
**Combustible tobacco use during Wave 2** (ref: not during Wave 2)	**2.589**	(1.301–5.151)**
**Days used e-cigarette in past 30 days**	1.003	(0.960–1.048)

* p<0.05

** p<0.01

*** p<0.001

## DISCUSSION

Appealing flavors was the most commonly reported motivation for using ENDS, but was mentioned more often among females. It is possible that flavors may be especially attractive to adolescent girls, as similar preferences for flavored products are observed in other products such as alcohol^16^. Perceptions of reduced harm to self and to others were the next most commonly reported reasons for ENDS use across all youth. This reason did not differ across sociodemographic groups, indicating that perceptions of reduced harm are important for most adolescent ENDS users, regardless of sociodemographic characteristics. Advertising was not commonly reported as a reason for ENDS use.

Logistic regression analyses revealed that similarity or exchangeability with cigarettes is an important reason for ENDS use among combustible tobacco users. Further research should explore whether ENDS are being used as an alternative to combustible tobacco among this group. More intense ENDS use was associated with reasons involving other people, indicating that social benefit may encourage more intense use.

These findings offer several insights that could be helpful for policy and practice. First, given flavor’s importance in motivating product use, limiting appealing flavors may be an important approach to reduce use among youth, particularly female youth. Second, measures to increase the price of ENDS may have more of an effect on those of other non-Hispanic ethnicities compared to non-Hispanic Whites, as this group had a higher likelihood of reporting affordability as a reason for ENDS use. Conversely, price-increasing measures may have less of an effect on females compared to males, as females were less likely to report affordability as a motivation for ENDS use. Additionally, given the high number of youth overall who report using ENDS because they believe they are less harmful to them than cigarettes, education campaigns should communicate the potential harms of ENDS. Finally, given the prevalence of youth reporting using ENDS because people who are close to them use them, campaigns or interventions that leverage the role of important people in youths’ lives may be a useful strategy.

Several limitations must be considered when interpreting our findings. First, individuals may be more likely to select those motivations that come quickly to mind. With no opportunity to report motivations outside those included in the PATH study questionnaire, other important motivations may not be reflected in the study and this analysis. Second, only youth who had used ENDS in the past 30 days were asked questions about motivations for use. Therefore, we cannot draw conclusions about motivations for use among individuals outside this group. Moreover, this sample size was relatively small. While weighting procedures were employed, future work should be done with larger samples. Finally, data on specific racial/ethnic groups other than non-Hispanic White, non-Hispanic Black and Hispanic were not available in the data file, so we are unable to draw conclusions about motivations for ENDS use among these specific populations.

## CONCLUSIONS

A long history of health disparities in tobacco product use^17^ underscores the need for tobacco control policies and interventions that effectively reduce ENDS use among vulnerable subpopulations of adolescents. Understanding the motivations for use among sociodemographic subgroups is an initial step towards informing the development of policies and interventions with equally distributed benefits.

## CONFLICTS OF INTEREST

The authors declare that they have no competing interests, financial or otherwise, related to the current work. M. B. Moran reports grants from National Institute on Drug Abuse/US Food and Drug Administration Center for Tobacco Products, during the conduct of the study. The rest of the authors have also completed and submitted an ICMJE form for disclosure of potential conflicts of interest.
